# Clinical validation of fully automated cartilage transverse relaxation time (T2) and thickness analysis using quantitative DESS magnetic resonance imaging

**DOI:** 10.1007/s10334-025-01227-5

**Published:** 2025-02-24

**Authors:** Wolfgang Wirth, Simon Herger, Susanne Maschek, Anna Wisser, Oliver Bieri, Felix Eckstein, Annegret Mündermann

**Affiliations:** 1 Chondrometrics GmbH, Freilassing, Germany; 2 Research Program for Musculoskeletal Imaging, Center for Anatomy and Cell Biology, Paracelsus Medical University, Strubergasse 21, 5020 Salzburg, Austria; 3 Ludwig-Boltzmann Institute for Arthritis and Rehabilitation, Paracelsus Medical University, Salzburg, Austria; 4 Department of Orthopedics and Traumatology, University Hospital Basel, Basel, Switzerland; 5 Department of Biomedical Engineering, University of Basel, Allschwil, Switzerland; 6 Department of Radiology, University Hospital Basel, Basel, Switzerland; 7 Department of Clinical Research, University of Basel, Basel, Switzerland

**Keywords:** Convolutional neural network, qDESS, Cartilage T2, Cartilage thickness, MRI

## Abstract

**Objective:**

To clinically validate a fully automated cartilage segmentation technique from quantitative double-echo steady-state (qDESS) MRI supporting simultaneous estimation of cartilage T2 and morphology. Here, we test whether laminar (superficial and deep layer) T2 results from convolutional neural network (CNN) segmentations are consistent with those from manual expert segmentations.

**Materials and methods:**

The 3D qDESS sequence was acquired using 3 T MRI (resolution: 0.3125 × 0.3125x1.5 mm) in both knees of 37 subjects with unilateral anterior cruciate ligament (ACL) injury and 48 uninjured controls. Automated femorotibial cartilage (FTJ) segmentation was based on a 2D U-Net. Laminar T2 and cartilage thickness across the FTJ) were compared between ACL-injured and contralateral knees, and between ACL-injured and control knees. Effect sizes of these differences were measured using non-parametric Cohen’s d (d_n-p_).

**Result:**

Significant differences were observed only in deep T2, with longer T2 in ACL-injured knees than in contralateral and healthy control knees in most of the comparisons and with similar effect sizes for automated and manual segmentations (range d_n-p_ automated/manual: 0.58–1.04/0.58–0.74). No significant differences were observed in superficial T2 or cartilage thickness.

**Discussion:**

Fully-automated, CNN-based analysis showed similar sensitivity to differences in laminar cartilage T2 as manual segmentation, allowing automated qDESS-analyses to be applied to larger datasets.

**Supplementary Information:**

The online version contains supplementary material available at 10.1007/s10334-025-01227-5.

## Introduction

Transverse (spin–spin) relaxation time (T2) analysis is frequently used for assessing alterations in cartilage hydration and collagen integrity and orientation related to early osteoarthritis (OA) [1]. These compositional changes of the cartilage matrix have been reported to result in prolonged cartilage T2 [2, 3], which has been associated with an increased risk of incident OA [4] or signs of mild OA (Kellgren–Lawrence (KL) grades 1–2) [1]. Cartilage T2 has typically been measured from 2D multi-echo spin–echo (MESE) MRI sequences [5], which provide an adequate spatial in-plane resolution for assessing laminar (superficial and deep layer) T2, but require relatively long acquisition times and exhibit greater partial volume effects than 3D MRI due to the larger slice thickness. Moreover, MESE MRI has been shown to not fully cover the deep articular cartilage [6].

3D quantitative double-echo at steady state (qDESS) is a variant of the commercially available DESS pulse sequence that retains both acquired echoes in contrast to the commercially available DESS pulse sequence, which merges the two echoes into a single mixed T1/T2 contrast image and discards the two acquired echoes immediately after acquisition [7, 8]. While the commercially available DESS sequence can be used for measuring cartilage morphology (thickness, volume, surface areas) with high resolution (unlike MESE) [9] and for semi-quantitative assessments of joint structural pathology [8, 10, 11], the qDESS sequence also allows the estimation of cartilage T2 from the two acquired (and retained) echoes [7, 8]. By extracting both cartilage T2 and morphology from the same set of cartilage segmentations in a single high-resolution MRI acquisition obtained with an efficient acquisition time, the qDESS sequence is more versatile and faster [8] than a conventional gradient echo sequence (for cartilage morphology) in combination with a MESE sequence (for cartilage T2), as for instance done in the Osteoarthritis Initiative protocol [12].

The MechSens trial [13] is the first clinical study to use the qDESS for the simultaneous quantitative analysis of cartilage morphology and T2. The primary aim of the MechSens trial was to investigate the dose–response relationship between ambulatory load and load-induced cartilage biomarker kinetics in participants with different OA risk. For this purpose, the MechSens trial enrolled participants with and without ACL injury in two different age groups [13]. Herger et al. [14] recently reported prolonged cartilage T2 in the deep cartilage layer of ACL-injured knees when compared to their contralateral uninjured knees and to knees from healthy controls. No significant differences were, however, observed for superficial T2 or cartilage thickness between ACL-injured and contralateral or healthy control knees [14]. These analyses were still relatively time consuming since the segmentation of the cartilage from the qDESS was obtained by manual analysis by experienced readers, followed by an additional quality control by an expert (manual analysis time: several hours per scan including quality control, depending on the skill of the reader, the number of slices to be analyzed, and on the complexity of the segmentations due to the level of pathology). Thus, scaling these to larger epidemiological or interventional studies would not be straight-forward and also rather expensive with regard to analysis time and costs.

To date, only two studies have evaluated the performance of CNN-based cartilage segmentation from qDESS MRI [15, 16]. These studies reported on the technical validity [15] or intra- and inter-day reproducibility [16], but not clinical validity, i.e., whether a difference observed between two clinically distinct groups can be reproduced by CNN-based cartilage segmentation.

Therefore, the objective of the current study was to clinically validate a CNN-based automated cartilage segmentation technique using qDESS obtained from the MechSens trial to evaluate whether cartilage relaxometry and morphometry results observed from manual segmentation [14] can be reproduced using fully automated CNN-based cartilage segmentation.

## Materials and methods

### Participants

The MechSens trial (ClinicalTrials.gov ID NCT04128566) recruited participants with a unilateral ACL injury that had occurred 2–10 years prior to enrollment, without concomitant or other knee injuries, and participants without any knee injury (controls). All participants were generally healthy and physically active (> 2 h of moderate-intensity aerobic activity per week), had a body mass index < 35 kg/m^2^ and no MRI contraindications (pacemaker, neurostimulator, pregnancy, metal splinters or metal prosthesis); further details on the demographics have been described previously [13, 14]. The MechSens trial aimed to recruit 96 participants across two age groups (20–30 and 40–60 years), with each age group comprising 12 women and 12 men with ACL injury, and 12 women and 12 men without knee injury. Of 89 participants who were recruited between January 2020 and December 2022, 85 had complete MRI baseline data (20–30 years ACL injury: *n* = 14/9 women/men, mean ± standard deviation age: 26.1 ± 3.1 years, BMI: 23.8 ± 2.8 kg/m^2^, time since injury: 5.2 ± 2.8 years; 20–30 years controls: *n* = 12/12, age: 26.6 ± 3.0 years, BMI: 22.9 ± 2.0 kg/m^2^; 40–60 years ACL injury: *n* = 10/4, age: 51.5 ± 5.5 years, BMI: 24.7 ± 2.6 kg/m^2^, time since injury: 5.6 ± 2.7 years; 40–60 years controls: *n* = 12/12, age: 49.0 ± 6.1 years, BMI: 24.7 ± 4.3 kg/m^2^) [13, 14]. Because the collection and processing of 2-year follow-up data is still ongoing, the current study only used baseline data. The MechSens trial was approved by the regional ethics board (Ethics Committee Northwest and Central Switzerland EKNZ 2019–01315) and conducted in accordance with the Declaration of Helsinki. All participants provided written informed consent.

### Imaging and manual segmentation

**Fig. 1:**
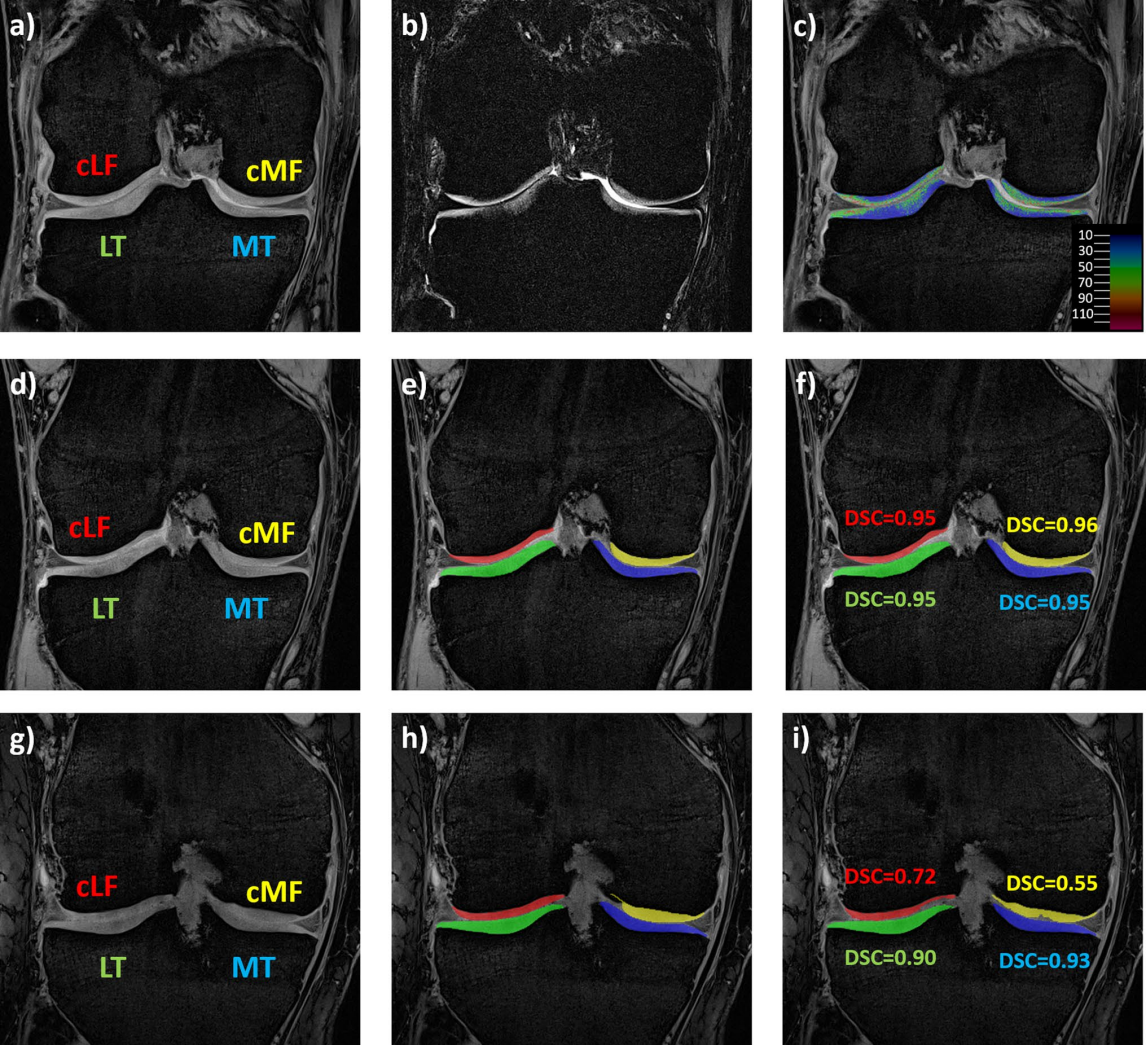
Illustration showing qDESS MRIs, cartilage T2 maps and the segmentation agreement. The top row shows the first echo (**a**), the second echo (**b**), and the cartilage T2 map together with the color code map (**c**, in ms). The middle row shows the 1st echo of the qDESS MRI (**d**), the manual segmentation (**e**), and the automated segmentation (**f**) in the knee with the highest segmentation agreement. The bottom row shows the 1st echo of the qDESS MRI (**g**), the manual segmentation (**h**), and the automated segmentation (**i**) in the knee with the lowest segmentation agreement. (DSC: Dice Similarity Coefficient)

The MechSens imaging and manual analysis protocol has been described in detail [14]. The imaging protocol included a double-oblique coronal qDESS sequence (in-plane resolution: 0.31 × 0.31 mm, acquisition matrix: 512 × 512, number of slices: 64–72, slice thickness: 1.5 mm, repetition/echo time: 17 ms/4.85 ms, flip angle: 15°, acquisition time: 9–12 min) that was acquired separately for both knees and covered the entire knee joint (Fig. 1). The qDESS was the only imaging sequence from the protocol [14] used for analyzing cartilage T2 and morphometry in the current study (Fig. 1) [7]. MRI scans of both knees were acquired after 20–30 min of sitting without weight-bearing using a 3 T Prisma scanner and a 15-channel Tx/Rx knee coil (both Siemens Healthineers, Erlangen, Germany). The software for the qDESS sequence was developed at the Department of Radiology, University Hospital Basel, Basel, Switzerland and was installed on the scanner (software version: VE11C) based on a research agreement with the manufacturer.

Manual segmentation of the cartilages in the weight-bearing femorotibial joint (MT & LT: medial & lateral tibia and cMF & cLF: central medial & lateral femoral condyle) was performed by experienced readers (Chondrometrics GmbH, Freilassing, Germany) blinded to injury status, sex, and age [14]. All segmentations were quality-controlled by an expert reader and corrections were made as needed.

### Automated cartilage segmentation

The CNN-based automated cartilage segmentation was based on a U-Net architecture previously adapted and validated for morphometric analysis of 3D gradient echo MRI [17, 18] and for cartilage T2 analysis of 2D MESE MRI [19, 20]. Because no separate set of qDESS MRIs was available for training the U-Net, two separate U-Nets were trained, one from both knees of 42 odd-numbered MechSens participants (training set: *n* = 70 knees from 35 participants; validation set: *n* = 14 knees from 7 participants) and one from both knees of 42 even-numbered MechSens participants (training set: *n* = 70 knees from 35 participants; validation set: *n* = 14 knees from 7 participants). Both U-Nets were trained on all MRI slices of both knees of the respective training sets. Left knees were flipped horizontally to ensure consistent location of medial and lateral cartilages in the coronal images. To account for the two qDESS echoes, the first convolutional layer of the U-Nets consisted of two input channels. The training was configured to use five labels with a weight of 0.22 for each of the four femorotibial cartilages, and a weight of 0.12 for the background (weights determined empirically). We also used a weighted cross-entropy loss function, Adam optimization with an initial learning rate of 0.01 and a mini-batch size of four slices per training iteration. Network weights were randomly initialized using a variance scaling initializer. The U-Nets were implemented in Python (Python Software Foundation, DE, USA) and used the TensorFlow framework (Google LLC, Mountain View, CA, USA).

The two independently trained U-Nets were then applied to the segmentation of the respective other group (even or odd-numbered) of participants (Fig. 1). After the automated segmentation, an automated post-processing was performed to detect and correct problems, such as filling of small enclosed unsegmented areas, eliminating implausible segmentations (e.g., fragments not connected to the main segmentation in the same or other slices), and smoothing of segmentation spikes. In contrast to the manual segmentation, no quality control or manual correction was performed for the automated cartilage segmentations. The automated analysis took approximately 26 s for each qDESS volume (1 s for the U-Net segmentation and 25 s for the post-processing).

### Cartilage T2 and thickness analysis

Cartilage T2 was estimated after acquisition using in-house software (Chondrometrics GmbH, Freilassing, Germany) for each of the segmented voxels as described previously [7, 14]. Briefly, T2 was calculated from the two steady-state free precession (SSFP) echoes (1st echo: S^+^, 2nd echo: S^−^) by numerically minimizing the difference between the observed signal ratio $\frac{{S}_{obs}^{-}}{{S}_{obs}^{+}}$ and the ratio between the analytically derived signal intensities $\frac{{{\text{S}}^{ - } \left( {\alpha ,TR,T_{1} ,T_{2} } \right)}}{{{\text{S}}^{ + } \left( {\alpha ,TR,T_{1} ,T_{2} } \right)}} \cdot e^{{\frac{{2 \cdot TE}}{{T_{2} }}}}$ (with $\frac{{{\text{S}}^{ - } \left( {\alpha ,TR,T_{1} ,T_{2} } \right)}}{{{\text{S}}^{ + } \left( {\alpha ,TR,T_{1} ,T_{2} } \right)}} \cdot e^{{\frac{{2 \cdot TE}}{{T_{2} }}}} = \frac{{1 - \left( {1 - E_{1} \cdot {\text{cos}}\alpha } \right) \cdot r}}{{1 - \left( {E_{1} - {\text{cos}}\alpha } \right) \cdot r}} \cdot e^{{\frac{{2 \cdot TE}}{{T_{2} }}}}$, ${E}_{1}= {e}^{-\frac{TR}{{T}_{1}}}$, $r = \left( {1 - E_{2}^{2} } \right) \cdot \left( {p^{2} - q^{2} } \right)^{{ - \frac{1}{2}}}$, ${E}_{2}= {e}^{-\frac{TR}{{T}_{2}}}$, $p=1-{E}_{1}cos \alpha -{E}_{2}^{2}\left({E}_{1}-cos \alpha \right)$, $q = E_{2} \left( {1 - E_{1} } \right)\left( {1 + \cos \alpha } \right)$, α = 15°, TR = 17 ms, TE = 4.85 ms, and T1 = 1000 ms, T1 estimate based on [21]). The numerical minimization was performed using a golden section search method, which was initialized with a T2 search range ranging from 0 to 5000 ms.

Because cartilage T2 is dependent on tissue depth [2], the four cartilage plates were divided into the top (superficial) and bottom (deep) 50%, based on the distance of each cartilage voxel to the cartilage surface and bone interface, respectively. Laminar T2 across the total femorotibial joint (FTJ) was derived as the average of all four femorotibial cartilages. Laminar T2 in the medial and lateral femorotibial compartment (MFTC & LFTC) was derived as the average T2 observed in the respective medial and lateral cartilages (MFTC: MT and cMF, LFTC: LT and cLF).

Cartilage thickness was computed for the four cartilage plates from the cartilage segmentations as described previously [22]. Cartilage thickness in the MFTC and LFTC was calculated as the sum of the cartilage thickness of the respective medial/lateral cartilages. Total FTJ cartilage thickness was calculated as the average of MFTC and LFTC cartilage thickness.

### Statistical analysis

Segmentation agreement between automated and manual cartilage segmentations was evaluated using the Dice similarity coefficient (DSC, range: 0 to 1) and the Hausdorff distance (HD), with the HD measuring the distance between border voxels of automated vs manual segmentations (in mm, 0 mm representing perfect overlap of border voxels). The accuracy of laminar cartilage T2 and cartilage thickness from automated vs. manual cartilage segmentations was evaluated using Bland & Altman plots. Intraclass correlation coefficients (ICC, two-way mixed effects, absolute agreement, single rater/measurement) were used to evaluate the correlation between automated vs. manual cartilage segmentations for laminar cartilage T2 and cartilage thickness, Pearson correlation coefficients are reported in Supplemental Table 1 to allow comparisons with other studies.

**Table 1:** Segmentation agreement between automated and manual cartilage segmentations (mean and standard deviation (SD) measured using the Dice Similarity Coefficient (DSC, range: 0 (no agreement) to 1 (perfect agreement)) and the Hausdorff Distance (HD, in mm) in the medial and lateral tibia (MT & LT) and the central medial and lateral femoral condyle (cMF & cLF)

	DSC		HD	
	Mean	SD	Mean	SD
MT	0.92	0.02	2.82	1.52
cMF	0.90	0.05	3.08	1.42
LT	0.93	0.02	3.11	1.15
cLF	0.90	0.04	2.53	1.04

Cross-sectional differences between ACL-injured and contralateral knees (between-knee comparison) and between ACL-injured and left and right knees from control participants (between-group comparison) were evaluated as previously done in the primary analysis [14], but now for both segmentation techniques. The primary focus of analysis was on comparing effect sizes of between-knee and between-group differences in laminar T2 and cartilage thickness in the total FTJ between segmentation techniques. Effect sizes of between-knee and between-group differences were also reported for the MFTC and LFTC for both segmentation techniques. Cartilage T2 and thickness measures were tested for outliers and the Shapiro–Wilk test and Q–Q plots were used to test for normality. Because the data of some of the groups were not normally distributed, results of between-knee and between-group comparisons were reported as medians and 25th/75th percentiles. Non-parametric tests were also used for detecting between-knee and between-group differences. Kruskal–Wallis tests were used to assess overall effects. Conovar–Iman (between-knee) and Dunn (between-group) tests were used for the post hoc comparisons. To account for multiple comparisons, the p values were adjusted using Holm correction (p_adj_), the significance level was p_adj_ < 0.05.

A non-parametric variant of the Cohen’s D (d_n-p_) was used for comparing effect sizes of between-knee and between-group comparisons between segmentation techniques. d_n-p_ was computed using the difference in the medians instead of the difference in the means, and using the pooled median absolute deviation instead of the pooled standard deviation. The statistical group comparisons were performed with the R package PMCMRplus (v1.9.6; Pohlert; 2022). The correlation analyses were performed using the R packages irr (v.0.84.1; Gamer et al.; 2019) and stats (v.3.5.2; R Core Team; 2023).

## Results

### Segmentation agreement and accuracy

Automated cartilage segmentation showed high agreement with manual segmentation (Table 1). The DSC was the lowest for the central medial and lateral weight-bearing femur (mean ± standard deviation, medial: 0.90 ± 0.05, lateral: 0.90 ± 0.04) and was the highest for the lateral tibia (0.93 ± 0.02, Table 1, Fig. 1).

**Fig. 2:**
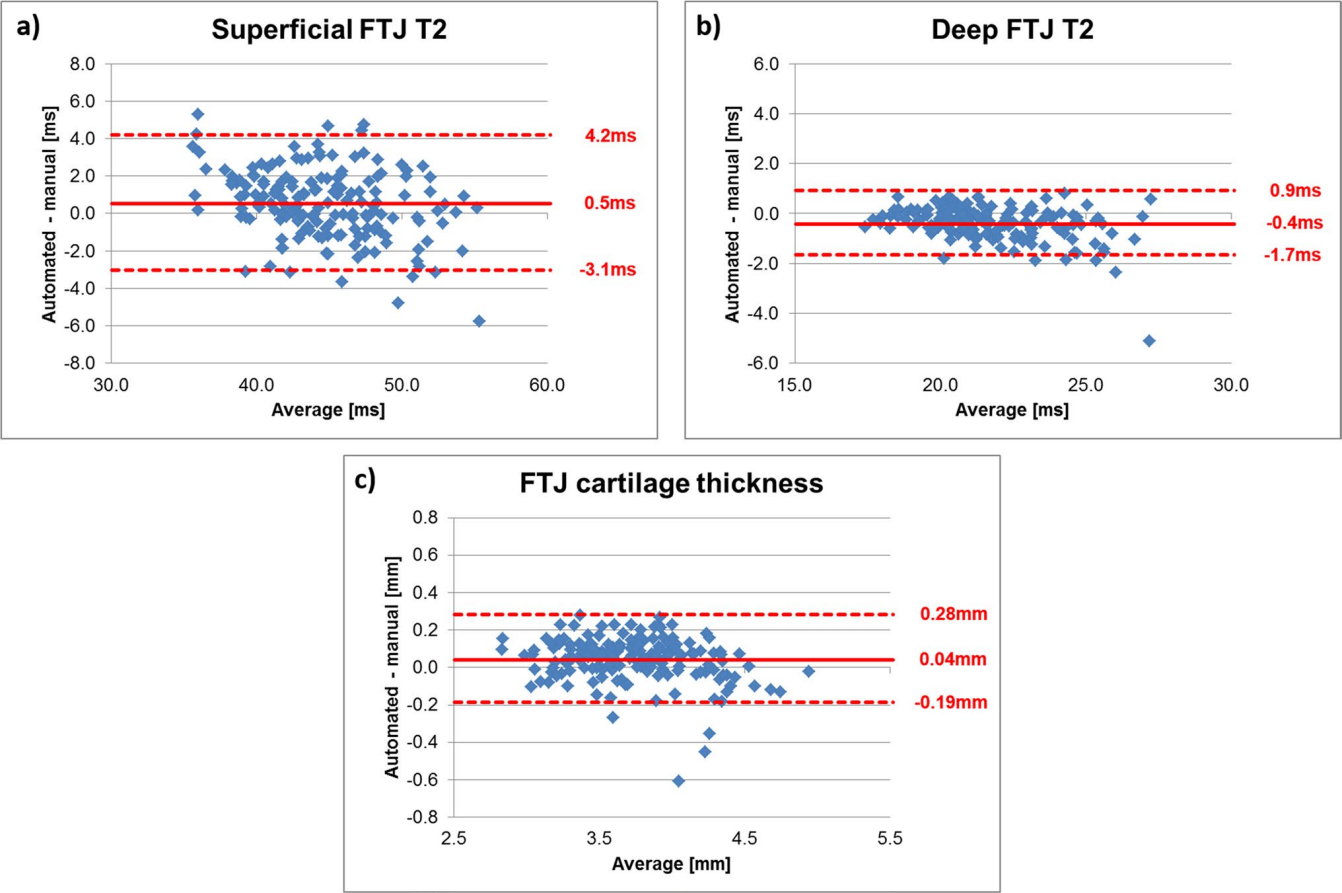
Bland & Altman plots comparing superficial (**a**) and deep layer T2 (**b**) and cartilage thickness (**c**) across the total femorotibial joint (FTJ) between automated and manual cartilage segmentations

Superficial FTJ T2 derived from automated segmentation tended to be longer than that derived from manual segmentation (mean ± standard deviation, 1.1 ± 4.2%, Fig. 2, Table 2). In contrast, deep automated FTJ T2 tended to be shorter than that derived from manual segmentation (− 1.9 ± 3.1%, Fig. 2, Table 2). The ICC between automated and manual segmentation T2 was 0.91 for superficial and 0.93 for deep FTJ T2 (Table 2, see Supplemental Table 1 for Pearson correlation). FTJ cartilage thickness derived from automated segmentation tended to overestimate that derived from manual segmentation (1.2 ± 3.2%), with an ICC of 0.95 (Table 2). Results for MFTC and LFTC were consistent with those observed for FTJ (Table 2).

**Table 2:** Accuracy and intraclass correlation coefficients (ICC) of superficial and deep layer T2 [ms] and cartilage thickness [mm] in the total femorotibial joint (FTJ), the medial (MFTC) and the lateral (LFTC) femorotibial compartment from automated vs. manual cartilage segmentations

		Automated segmentation		Manual segmentation		Accuracy (mean difference)		ICC (95% CI)
		Mean	SD	Mean	SD	Mean	SD	
Superficial layer	FTJ	44.9	4.2	44.4	4.7	0.5	1.9	0.91 (0.87, 0.93)
	MFTC	44.2	5.4	44.1	6.0	0.1	2.3	0.92 (0.89, 0.94)
	LFTC	45.6	4.1	44.7	4.7	0.9	2.0	0.87 (0.79, 0.92)
Deep layer	FTJ	21.4	2.0	21.8	2.2	− 0.4	0.7	0.93 (0.84, 0.96)
	MFTC	21.1	2.1	21.6	2.4	− 0.5	0.8	0.91 (0.79, 0.95)
	LFTC	21.8	2.2	22.1	2.4	− 0.3	0.7	0.94 (0.90, 0.96)
Cartilage thickness	FTJ	3.75	0.39	3.71	0.42	0.0	0.1	0.95 (0.93, 0.97)
	MFTC	3.57	0.41	3.54	0.46	0.0	0.2	0.93 (0.91, 0.95)
	LFTC	3.93	0.44	3.88	0.45	0.1	0.1	0.96 (0.93, 0.97)

*SD* standard deviation, *95% CI* 95% confidence intervals

### Between-knee and between-group comparisons

Superficial layer T2 and cartilage thickness in the total FTJ, MFTC, and LFTC did not differ between ACL-injured and uninjured contralateral knees, or between ACL-injured and healthy control knees, for both automated and manual segmentations and for both age groups (Tables 3 & 4). The effect sizes for most of these comparisons were in the same range for automated and manual segmentations (Tables 3 & 4).

**Table 3:** Comparison of superficial and deep layer T2 [ms] and cartilage thickness [mm] between ACL-injured and contralateral knees obtained from automated and manual cartilage segmentations

		ACL-injured		Contralateral		d_n-p_	p_adj_
		Median	IQR	Median	IQR		
Age 20–30 years, manual							
Cartilage thickness	FTJ	3.93	(3.62, 4.17)	3.78	(3.60, 3.89)	0.31	0.593
	MFTC	3.77	(3.28, 3.97)	3.52	(3.32, 3.72)	0.37	0.235
	LFTC	4.09	(3.77, 4.45)	3.98	(3.65, 4.17)	0.15	0.235
Superficial T2	FTJ	43.6	(39.4, 47.7)	43.4	(39.4, 44.8)	0.02	1.000
	MFTC	42.2	(38.7, 46.7)	42.8	(38.2, 46.1)	− 0.07	1.000
	LFTC	44.5	(41.3, 47.8)	42.5	(40.7, 44.7)	0.30	1.000
Deep T2	FTJ	22.8	(21.1, 24.3)	21.1	(20.0, 21.8)	0.58	< 0.001
	MFTC	22.3	(21.4, 23.6)	20.9	(20.2, 21.4)	0.70	0.333
	LFTC	23.9	(21.4, 25.0)	21.2	(20.1, 22.1)	0.83	0.012
Age 20–30 years, automated							
Cartilage thickness	FTJ	3.90	(3.63, 4.17)	3.84	(3.64, 4.00)	0.12	0.235
	MFTC	3.71	(3.45, 3.93)	3.62	(3.42, 3.74)	0.19	0.593
	LFTC	4.04	(3.80, 4.51)	4.07	(3.78, 4.22)	− 0.04	0.684
Superficial T2	FTJ	42.5	(40.2, 47.4)	43.7	(40.9, 45.7)	− 0.19	1.000
	MFTC	41.3	(38.6, 46.3)	42.2	(39.6, 45.8)	− 0.13	1.000
	LFTC	44.4	(42.6, 47.4)	44.0	(42.2, 45.4)	0.08	1.000
Deep T2	FTJ	22.4	(20.8, 23.9)	20.5	(19.9, 21.3)	0.68	< 0.001
	MFTC	21.9	(20.7, 23.3)	20.2	(19.5, 20.9)	0.75	< 0.001
	LFTC	23.5	(20.8, 24.5)	21.0	(19.8, 21.7)	0.92	0.002
Age 40–60 years, manual							
Cartilage thickness	FTJ	3.50	(3.22, 4.35)	3.49	(3.27, 3.91)	0.02	0.684
	MFTC	3.43	(3.05, 4.17)	3.29	(3.15, 3.85)	0.18	1.000
	LFTC	3.62	(3.43, 4.37)	3.66	(3.51, 4.02)	− 0.07	0.906
Superficial T2	FTJ	44.0	(42.4, 48.6)	46.0	(43.1, 50.5)	− 0.26	1.000
	MFTC	42.5	(39.3, 51.7)	45.8	(42.8, 50.6)	− 0.27	1.000
	LFTC	45.6	(44.2, 48.2)	46.4	(41.1, 48.9)	− 0.11	1.000
Deep T2	FTJ	24.1	(23.0, 25.5)	22.0	(20.3, 23.6)	0.61	0.078
	MFTC	23.4	(21.5, 24.9)	21.1	(20.3, 24.2)	0.58	< 0.001
	LFTC	25.5	(24.2, 26.6)	22.0	(20.7, 24.6)	1.02	< 0.001
Age 40–60 years, automated							
Cartilage thickness	FTJ	3.50	(3.33, 4.05)	3.55	(3.38, 3.89)	− 0.08	0.852
	MFTC	3.36	(3.15, 3.88)	3.39	(3.13, 3.85)	− 0.04	1.000
	LFTC	3.72	(3.44, 4.22)	3.72	(3.65, 4.00)	0.00	0.444
Superficial T2	FTJ	44.0	(42.7, 46.8)	46.5	(43.0, 50.7)	− 0.36	1.000
	MFTC	44.2	(39.4, 48.3)	45.4	(43.6, 49.4)	− 0.14	1.000
	LFTC	45.2	(43.7, 47.0)	46.1	(42.0, 49.3)	− 0.12	1.000
Deep T2	FTJ	23.8	(22.3, 24.9)	21.1	(20.4, 23.4)	1.04	0.008
	MFTC	22.7	(21.0, 24.1)	20.5	(20.0, 23.4)	0.59	0.008
	LFTC	24.3	(22.7, 26.1)	22.0	(21.1, 23.4)	0.73	0.222

*IQR* inter-quartile range, *d*_*n-p*_ non-parametric d (measure of effect size), *FTJ* femorotibial joint, *MFTC/LFTC* medial/lateral femorotibial compartment, *p*_*adj*_ p-values adjusted for multiple comparisons

**Table 4:** Comparison of superficial and deep layer T2 [ms] and cartilage thickness [mm] between ACL-injured and left & right control knees obtained from automated and manual cartilage segmentations

		ACL-injured		Healthy_left_				Healthy_right_			
		Median	IQR	Median	IQR	d_n-p_	p_adj_	Median	IQR	d_n-p_	p_adj_
Age 20–30 years, manual											
Cartilage thickness	FTJ	3.93	(3.62, 4.17)	3.60	(3.40, 3.82)	0.64	0.771	3.67	(3.52, 3.83)	0.54	1.000
	MFTC	3.77	(3.28, 3.97)	3.38	(3.24, 3.75)	0.54	1.000	3.46	(3.19, 3.74)	0.42	1.000
	LFTC	4.09	(3.77, 4.45)	3.77	(3.54, 4.17)	0.45	1.000	3.90	(3.60, 4.02)	0.27	1.000
Superficial T2	FTJ	43.6	(39.4, 47.7)	44.2	(40.9, 47.2)	− 0.07	1.000	43.5	(41.2, 47.1)	0.02	1.000
	MFTC	42.2	(38.7, 46.7)	44.0	(40.4, 49.1)	− 0.19	1.000	43.2	(40.4, 45.4)	− 0.14	1.000
	LFTC	44.5	(41.3, 47.8)	44.7	(41.7, 46.6)	− 0.02	1.000	44.4	(41.6, 46.5)	0.01	1.000
Deep T2	FTJ	22.8	(21.1, 24.3)	20.8	(20.3, 21.4)	0.74	0.044	21.1	(20.2, 21.7)	0.61	0.055
	MFTC	22.3	(21.4, 23.6)	20.7	(19.9, 21.4)	0.72	0.086	20.6	(19.8, 21.6)	0.77	0.069
	LFTC	23.9	(21.4, 25.0)	21.1	(20.5, 21.8)	0.93	0.160	21.0	(20.4, 22.4)	0.90	0.161
Age 20–30 years, automated											
Cartilage thickness	FTJ	3.90	(3.63, 4.17)	3.62	(3.48, 3.86)	0.52	1.000	3.76	(3.61, 3.88)	0.30	1.000
	MFTC	3.71	(3.45, 3.93)	3.45	(3.25, 3.76)	0.49	1.000	3.58	(3.27, 3.72)	0.23	1.000
	LFTC	4.04	(3.80, 4.51)	3.74	(3.63, 4.17)	0.40	1.000	3.97	(3.72, 4.14)	0.10	1.000
Superficial T2	FTJ	42.5	(40.2, 47.4)	44.1	(42.5, 47.6)	− 0.28	1.000	44.7	(42.5, 47.7)	− 0.35	1.000
	MFTC	41.3	(38.6, 46.3)	43.4	(40.2, 48.5)	− 0.26	1.000	43.5	(41.2, 45.6)	− 0.35	1.000
	LFTC	44.4	(42.6, 47.4)	45.5	(43.1, 47.1)	− 0.19	1.000	45.5	(44.1, 49.5)	− 0.20	1.000
Deep T2	FTJ	22.4	(20.8, 23.9)	20.6	(20.0, 21.3)	0.69	0.046	20.8	(20.4, 21.5)	0.60	0.222
	MFTC	21.9	(20.7, 23.3)	20.5	(19.6, 21.4)	0.62	0.301	20.6	(19.9, 21.3)	0.61	0.366
	LFTC	23.5	(20.8, 24.5)	21.1	(20.2, 21.6)	0.90	0.079	20.7	(20.3, 22.1)	1.01	0.169
Age 40–60 years, manual											
Cartilage thickness	FTJ	3.50	(3.22, 4.35)	3.67	(3.33, 3.95)	− 0.24	1.000	3.70	(3.33, 3.93)	− 0.28	1.000
	MFTC	3.43	(3.05, 4.17)	3.54	(3.19, 3.67)	− 0.16	1.000	3.57	(3.19, 3.74)	− 0.18	1.000
	LFTC	3.62	(3.43, 4.37)	3.82	(3.37, 4.21)	− 0.24	1.000	3.67	(3.39, 4.11)	− 0.07	1.000
Superficial T2	FTJ	44.0	(42.4, 48.6)	44.4	(40.8, 48.7)	− 0.05	1.000	44.7	(42.5, 47.0)	− 0.13	1.000
	MFTC	42.5	(39.3, 51.7)	44.2	(39.5, 49.6)	− 0.14	1.000	44.2	(41.2, 46.8)	− 0.16	1.000
	LFTC	45.6	(44.2, 48.2)	44.2	(41.7, 47.0)	0.26	1.000	44.9	(41.9, 47.2)	0.13	1.000
Deep T2	FTJ	24.1	(23.0, 25.5)	21.1	(19.5, 24.5)	0.65	0.036	21.7	(19.4, 23.7)	0.58	0.029
	MFTC	23.4	(21.5, 24.9)	21.2	(19.3, 23.5)	0.49	0.609	21.4	(19.7, 23.4)	0.47	0.625
	LFTC	25.5	(24.2, 26.6)	21.2	(19.9, 24.2)	0.95	0.013	21.5	(19.5, 23.9)	0.91	0.004
Age 40–60 years, automated											
Cartilage thickness	FTJ	3.50	(3.33, 4.05)	3.70	(3.38, 4.03)	− 0.27	1.000	3.79	(3.39, 4.04)	− 0.37	1.000
	MFTC	3.36	(3.15, 3.88)	3.55	(3.33, 3.76)	− 0.31	1.000	3.65	(3.29, 3.84)	− 0.38	1.000
	LFTC	3.72	(3.44, 4.22)	3.86	(3.38, 4.26)	− 0.15	1.000	3.85	(3.49, 4.19)	− 0.17	1.000
Superficial T2	FTJ	44.0	(42.7, 46.8)	45.3	(42.2, 49.4)	− 0.18	1.000	46.0	(42.7, 47.9)	− 0.35	1.000
	MFTC	44.2	(39.4, 48.3)	44.9	(40.9, 49.2)	− 0.07	1.000	44.8	(41.2, 48.5)	− 0.06	1.000
	LFTC	45.2	(43.7, 47.0)	45.4	(42.8, 49.1)	− 0.03	1.000	45.9	(43.1, 48.5)	− 0.14	1.000
Deep T2	FTJ	23.8	(22.3, 24.9)	20.9	(19.5, 23.7)	0.72	0.100	21.4	(19.1, 23.0)	0.64	0.088
	MFTC	22.7	(21.0, 24.1)	20.9	(19.1, 23.1)	0.39	1.000	21.2	(19.2, 22.7)	0.34	1.000
	LFTC	24.3	(22.7, 26.1)	21.3	(20.0, 24.2)	0.67	0.041	21.5	(19.3, 23.0)	0.63	0.010

*IQR* inter-quartile range, *d*_*n-p*_ non-parametric d (measure of effect size), *FTJ* femorotibial joint, *MFTC/LFTC* medial/lateral femorotibial compartment, *p*_*adj*_ p-values adjusted for multiple comparisons

**Fig. 3:**
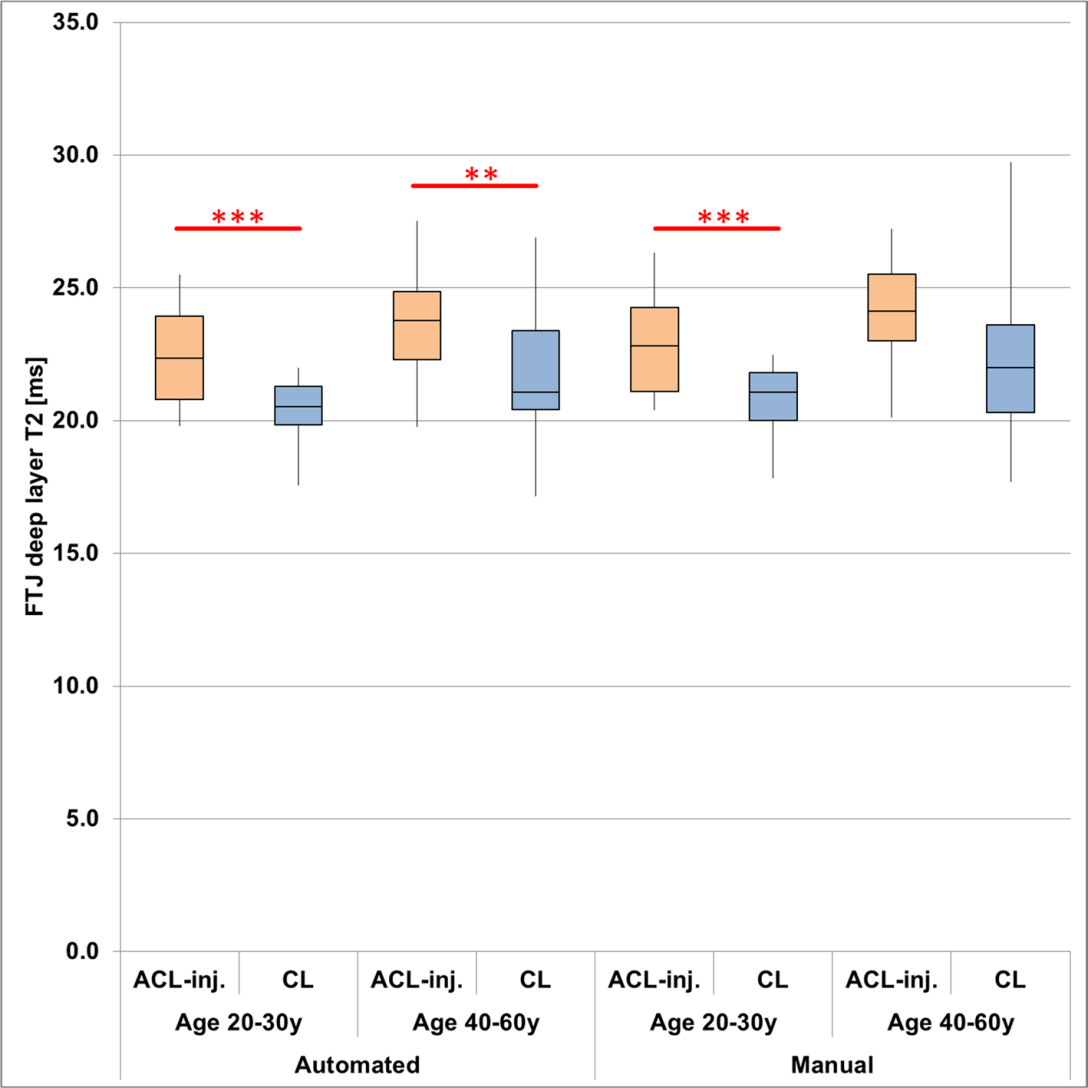
Boxplots of deep layer T2 across the total femorotibial joint (FTJ) obtained from manual and automated cartilage segmentations for ACL-injured (ACJ-inj) knees and contralateral (CL) knees without injury. The whiskers indicate the minimum and maximum values observed in each of the groups. (*: adjusted* p*<0.05; **: adjusted* p*<0.01; ***: adjusted* p*<0.001)

In the group of young ACL subjects, deep FTJ T2 was longer in the ACL-injured than in the uninjured contralateral knees both for automated (p_adj_ < 0.001) and manual segmentations (p_adj_ < 0.001) with similar effect sizes observed for both segmentation techniques (d_n-p_ automated/manual = 0.68/0.58, Fig. 3, Table 3). In the group of older ACL subjects, deep layer T2 was longer in the ACL-injured than in the uninjured contralateral knees when derived from automated (p_adj_ = 0.008) but not when derived from manual segmentations (p_adj_ = 0.078), and the effect size tended to be greater for automated (d_n-p_ = 1.04) than for manual cartilage segmentations (d_n-p_ = 0.61, Fig. 3, Table 3). Results for MFTC and LFTC are shown in Table 3.

**Fig. 4:**
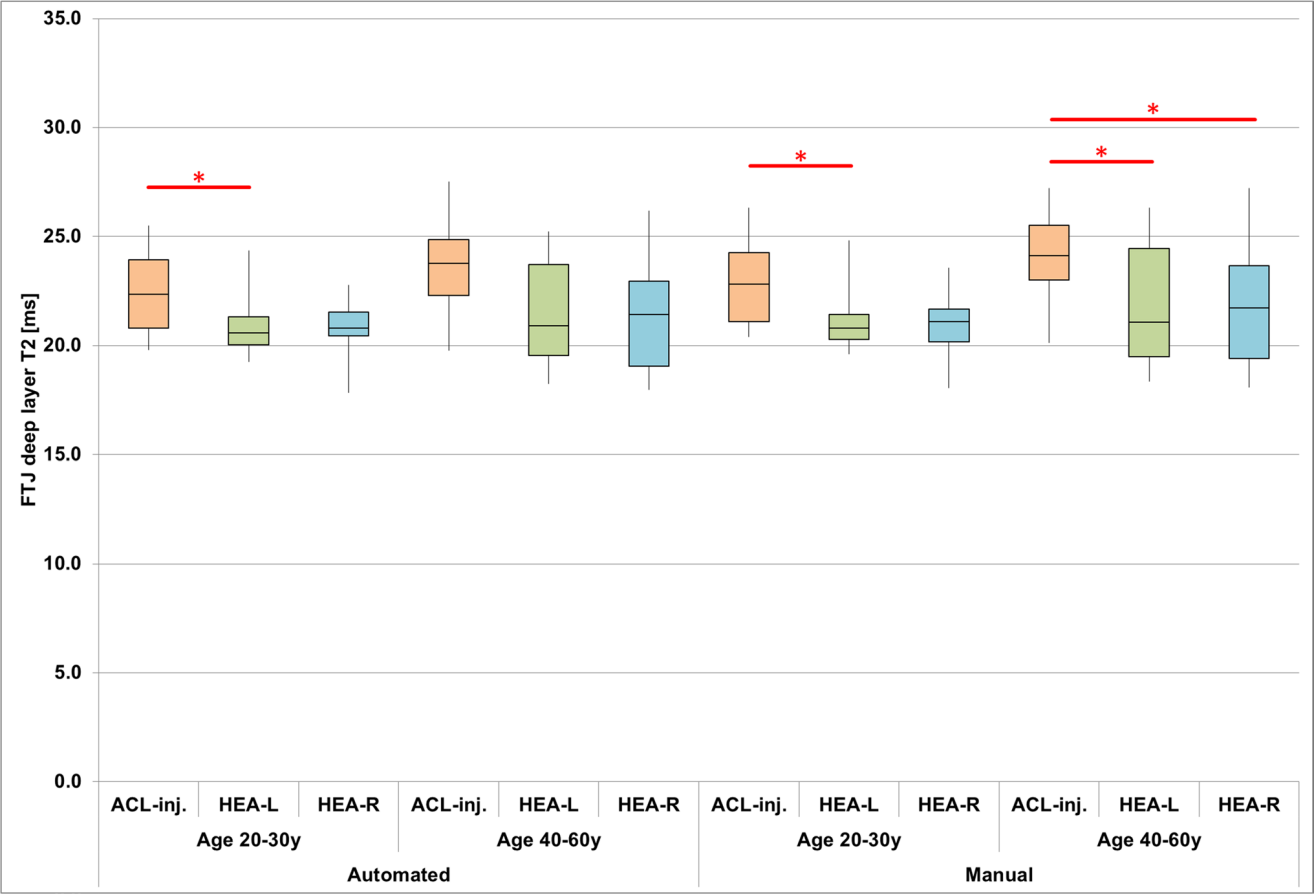
Boxplots of deep layer T2 across the total femorotibial joint (FTJ) obtained from manual and automated cartilage segmentations for ACL-injured (ACL-inj) knees and left (HEA-L) and right (HEA-R) knees from healthy controls without any injury. The whiskers indicate the minimum and maximum values observed in each of the groups. (*: adjusted* p*<0.05; **: adjusted* p*<0.01; ***: adjusted* p*<0.001)

In the younger age group, deep layer FTJ T2 was longer in ACL-injured knees than in left (but not right) knees from the younger control group for both automated (p_adj_ = 0.046) and manual cartilage segmentations (p_adj_ = 0.044), with similar effect sizes for both segmentation techniques (automated/manual: d_n-p_ = 0.69/0.74, Fig. 4, Table 4). The effect sizes for the right control knees were slightly lower than those for the left control knees and also similar between segmentation techniques (automated/manual: d_n-p_ = 0.60/0.61, Fig. 4, Table 4). In the older age group, deep layer FTJ T2 was longer for manual segmentations in ACL-injured than in both left and right knees from the older control group (left/right: p_adj_ = 0.036/0.029), whereas differences derived from automated cartilage segmentations were not statistically significant (left/right: p = 0.010/0.088, Fig. 4, Table 4). The effect sizes were, however, similar for automated (left/right: d_n-p_ = 0.72/0.64) and manual cartilage segmentations (left/right: d_n-p_ = 0.65/0.58, Fig. 4, Table 4). Please see Table 4 for results for MFTC and LFTC.

## Discussion

This is the first study to present data from a clinical validation of a CNN-based automated cartilage segmentation technique for high-resolution qDESS, an MRI sequence that permits obtaining both cartilage T2 and thickness measures from a single acquisition and single set of cartilage segmentations. We used a clinical model of ACL-injured knees that were compared with both contralateral uninjured and with healthy control knees, in which differences in deep layer cartilage T2, but not in superficial layer T2, and not in cartilage thickness were previously identified based on quality-controlled, manual gold standard segmentations [14]. The results of the between-knee and between-group comparisons obtained by automated segmentation were largely consistent with those from manual segmentation, with similar effect sizes for deep layer T2 for both segmentation techniques. The CNN-based cartilage segmentation technique applied to qDESS MRI therefore appears to be clinically valid and suitable for application to larger pre- or early OA cohorts.

The strengths of this study include the well-defined sample enrolled in MechSens, the well-controlled protocol, the availability of both uninjured contralateral knees and both knees from healthy subjects as controls, the availability of state-of-the-art, high-quality qDESS MRIs, and the availability of quality-controlled manual cartilage segmentations that were used for training the U-Nets and for providing manual reference values for this study. One limitation of the current study is the cross-sectional comparison of differences between an automated vs. manual segmentation technique since collection of longitudinal follow-up data is still ongoing. It will have to be seen whether the high accuracy observed in the current study also translates into a high sensitivity to differences in longitudinal change over time, once the collection of 24-month follow-up data has been completed. Another limitation is the relatively small size of the two ACL groups, which was mainly due to the exclusion of ACL patients with concomitant or other injuries [13, 14]. Still, the sample size was sufficient to detect differences between ACL-injured and non-injured knees even when using less sensitive non-parametric statistical methods, and when adjusting for multiple parallel comparisons. A numerical minimization approach was chosen for estimating T2 from the qDESS MRIs because it has been validated for estimating T2 from qDESS MRIs acquired with the qDESS implementation used in the current study [7]. More recently, a simple analytical approach has been proposed by Sveinsson et al. that is computationally more efficient than numerical minimization [23] and could potentially also be used for the qDESS implementation used in the current study after the required validation. Another limitation is the lack of test–retest acquisitions for this cohort, which precluded an analysis of the test–retest reproducibility of both segmentation techniques in this cohort. However, a previous study reported an excellent repeatability of qDESS MRI for cartilage T2 (coefficient of variation: 1.8%) and thickness analyses (coefficient of variation: 0.7%) [8], indicating that qDESS MRI is suitable for both cartilage relaxometry and morphometry. Finally, the U-Nets trained on data from the MechSens trial can most likely not be directly applied to qDESS MRIs acquired in other studies using different MRI equipment. Still, the data could be used for training combined models comprising qDESS MRIs from different scanners or could serve as basis for a model trained on different MRI characteristics after a transfer learning step.

MESE MRI has been shown to cover only about two-thirds of the cartilage depth covered by gradient echo MRI sequences such as DESS, missing a substantial part of the radial zone of the cartilage [6]. A recent study on ACL-injured participants performed using MESE MRI (at 1.5 T) and also using both automated and manual segmentation (same readers) did not observe T2 differences compared to healthy reference knees [19]. Since no differences were found in either the deep or in the superficial cartilage lamina with MESE MRI [19], qDESS MRI may be more sensitive than MESE MRI in detecting deep layer T2 alterations in early OA in ACL-injured patients, possibly due to its ability to cover the full radial zone of articular cartilage. Moreover, the qDESS sequence acquired in the MechSens trial also provided a better contrast and a better overall image quality than MESE MRIs typically acquired in OA studies. This likely explains the high segmentation agreement and high accuracy between automated and manual analyses observed in the current study, which exceeded that previously observed for MESE [19, 20, 24, 25].

The segmentation agreement observed in our study was in the same range as that previously observed for 3D FLASH and DESS MRI from the Osteoarthritis Initiative (OAI) using a similar image analysis pipeline [17], and the accuracy observed for cartilage thickness parameters was even slightly higher than that reported for OAI DESS MRI [17, 18, 26]. The observed agreement between automated and manual cartilage segmentations was also higher than that reported for qDESS by Schmidt et al. [15] potentially because Schmidt et al. included qDESS from different MRI scanners from different vendors, whereas the MRIs analyzed in our study were all acquired on the same scanner. In addition, Schmidt et al. trained the U-Net on the combined echo rather than exploiting the information from the two echoes [15]. Despite the greater agreement, the accuracy of the T2 measurements in our study was in the same range as that reported by Schmidt et al. [15], indicating that T2 measurements from qDESS are not highly sensitive to small segmentation errors. Williams et al. recently investigated the repeatability of CNN-based automated cartilage T2 analyses from qDESS and reported a test–retest precision of less than 1.3 ms, and an intra- and inter-day variability (coefficient of variation) of less than 5% [16]. However, neither Schmidt et al. nor Williams et al. reproduced a clinical effect using automated qDESS cartilage segmentation [15, 16], and hence did not clinically validate automated qDESS-based relaxometry or morphometry analyses.

In the MechSens trial, between-knee and between-group differences were observed only in deep but not in superficial layer T2 and cartilage thickness [14]. The lack of differences in cartilage thickness is typical for pre-OA or early OA knees and may be explained in this study by the still relatively short time since injury in the context of OA (2 to 10 years) and the simultaneous cartilage thinning and thickening previously reported in the years after the injury [27] and for knees with early radiographic OA [28]. The absence of superficial layer T2 differences is consistent with results of a previous study that investigated laminar T2 after ACL injury and reported that only deep T2 was increased in ACL-injured knees [29]. Nevertheless, for deep T2, the CNN-based automated analysis technique reproduced the between-knee and between-group differences previously observed in the MechSens trial using manual cartilage segmentation [14], with similar effect sizes for both segmentation techniques. The finding of differences in deep layer T2 between ACL-injured and contralateral knees in the older cohort in the automated analysis but not in our previous manual analysis [14] suggests that the CNN segmentation technology could potentially be more sensitive to these T2 changes than conventional segmentation approaches. However, the effect sizes observed in this study were still largely comparable between both segmentation techniques. The automated analysis of both laminar cartilage T2 and cartilage thickness from qDESS MRI therefore appears not only to be technically valid as shown by the high agreement and accuracy observed in this and also in previous studies [15, 16] but also to be clinically valid.

Current studies on OA are increasingly relying on automated cartilage segmentation for reducing analysis time and cost. Because of the excellent automated image analysis results that can be achieved with the qDESS and because of its ability to perform morphometry and relaxometry within a single acquisition [15], this MRI sequence should be considered a frontrunner in future imaging protocols although it may still take some time until it becomes commercially available on all relevant clinical MRI scanners. In addition, the qDESS allows to study knees at all stages of the disease. The MechSens trial demonstrated its sensitivity to differences in laminar T2, and the DESS sequence family has previously been shown to be sensitive (cross-sectionally and longitudinally) to differences in cartilage thickness in knees with moderate to advanced, radiographic OA [18, 30]. Therefore, the use of the qDESS may not only reduce the cost of the analysis, but also reduce the scan time and thus patient burden and cost of the MRI acquisition. A state-of-the-art imaging protocol that includes qDESS MRI and clinical sequences for a comprehensive analysis of joint tissues and pathology may require < 30 min per knee [14, 31].

## Conclusion

The fully automated CNN-based laminar cartilage T2 and cartilage thickness analysis showed high agreement and accuracy, translating into similar sensitivity to clinical differences in laminar T2, as previously observed with manual cartilage segmentations. A CNN-based image analysis workflow trained using high-quality cartilage segmentations may thus allow to replace manual cartilage segmentation in future studies using qDESS MRI, and may allow compositional cartilage analyses to be applied to datasets from larger cohorts.

## Supplementary Information

Below is the link to the electronic supplementary material.

Supplementary file1 (PDF 9 KB)

## Data Availability

Data will be made available upon study completion on reasonable request for scientific purposes.
