# Correction: Clinical validation of fully automated cartilage transverse relaxation time (T2) and thickness analysis using quantitative DESS magnetic resonance imaging

**DOI:** 10.1007/s10334-025-01255-1

**Published:** 2025-04-29

**Authors:** Wolfgang Wirth, Simon Herger, Susanne Maschek, Anna Wisser, Oliver Bieri, Felix Eckstein, Annegret Mündermann

**Affiliations:** 1 Chondrometrics GmbH, Freilassing, Germany; 2 Research Program for Musculoskeletal Imaging, Center for Anatomy and Cell Biology, Paracelsus Medical University, Strubergasse 21, 5020 Salzburg, Austria; 3 Ludwig-Boltzmann Institute for Arthritis and Rehabilitation, Paracelsus Medical University, Salzburg, Austria; 4 Department of Orthopedics and Traumatology, University Hospital Basel, Basel, Switzerland; 5 Department of Biomedical Engineering, University of Basel, Allschwil, Switzerland; 6 Department of Radiology, University Hospital Basel, Basel, Switzerland; 7 Department of Clinical Research, University of Basel, Basel, Switzerland

**Correction: Magnetic Resonance Materials in Physics, Biology and Medicine (2025) 38:285–297** 10.1007/s10334-025-01227-5

In the original version of this article, the ORCiD 0000-0001-5207-2100 for Simon Herger, ORCiD 0000-0002-6472-1689 for Annegret Muendermann, ORCiD 0000-0002-6755-5495 for Oliver Bieri and ORCiD 0000-0003-4068-3515 for Anna Wisser were inadvertently omitted.

