# Supplementary material for: Clinical validation of fully automated cartilage transverse relaxation time (T2) and thickness analysis using quantitative DESS magnetic resonance imaging
Source: MAGMA. 2025 Feb 24;38(2):285–97. doi: 10.1007/s10334-025-01227-5 (PMC11914229; doi:10.1007/s10334-025-01227-5)
Supplement: Supplementary file 1 — Supplementary file1 (PDF 9 KB) [file 10334_2025_1227_MOESM1_ESM.pdf]

Supplemental Table 1: Pearson correlation (r) of superficial and deep layer T2 and cartilage thickness in the total femorotibial joint (FTJ), the medial (MFTC) and the lateral (LFTC) femorotibial compartment from automated vs. manual cartilage segmentations

		r (95% CI)
**Superficial layer**	**FTJ**	0.92 (0.89, 0.94)
	**MFTC**	0.92 (0.90, 0.94)
	**LFTC**	0.90 (0.86, 0.92)
**Deep layer**	**FTJ**	0.96 (0.94, 0.97)
	**MFTC**	0.94 (0.92, 0.96)
	**LFTC**	0.96 (0.94, 0.97)
**Cartilage thickness**	**FTJ**	0.96 (0.94, 0.97)
	**MFTC**	0.94 (0.92, 0.96)
	**LFTC**	0.96 (0.95, 0.97)

r: Pearson correlation, 95% CI: 95% confidence intervals
